# Toward a biomarker panel measured in CNS-originating extracellular vesicles for improved differential diagnosis of Parkinson’s disease and multiple system atrophy

**DOI:** 10.1186/s40035-023-00346-0

**Published:** 2023-03-20

**Authors:** Hash Brown Taha, Simon Hornung, Suman Dutta, Leony Fenwick, Otmane Lahgui, Kathryn Howe, Nour Elabed, Irish del Rosario, Darice Y. Wong, Aline Duarte Folle, Daniela Markovic, Jose-Alberto Palma, Un Jung Kang, Roy N. Alcalay, Miriam Sklerov, Horacio Kaufmann, Brent L. Fogel, Jeff M. Bronstein, Beate Ritz, Gal Bitan

**Affiliations:** 1 Department of Neurology, David Geffen School of Medicine, University of California Los Angeles, Los Angeles, CA 90095 USA; 2 Department of Integrative Biology and Physiology, University of California Los Angeles, Los Angeles, CA 90095 USA; 3 Department of Epidemiology, Fielding School of Public Health, University of California Los Angeles, Los Angeles, CA 90095 USA; 4 Department of Medicine Statistics Core, Division of General Internal Medicine and Health Services Research, University of California Los Angeles, Los Angeles, CA 90095 USA; 5 Department of Neurology, Dysautonomia Center, New York University School of Medicine, New York, NY 10016 USA; 6 Department of Neurology, Taub Institute for Research on Alzheimer’s Disease and the Aging Brain, Columbia University, New York, NY 10032 USA; 7 Department of Neurology, Tel Aviv Sourasky Medical Center, Tel Aviv, Israel; 8 Department of Neurology, University of North Carolina School of Medicine, Chapel Hill, NC 27599 USA; 9 Department of Neurology, The Marlene and Paolo Fresco Institute for Parkinson’s and Movement Disorders, New York University School of Medicine, New York, NY 10016 USA; 10 Department of Human Genetics, David Geffen School of Medicine, University of California Los Angeles, Los Angeles, CA 90095 USA; 11 UCLA Clinical Neurogenomics Research Center, David Geffen School of Medicine, University of California, Los Angeles, CA 90095 USA; 12 Brain Research Institute, University of California, Los Angeles, CA 90095 USA; 13 Molecular Biology Institute, University of California Los Angeles, Los Angeles, CA 90095 USA; 14 Present Address: Division of Peptide Biochemistry, TUM School of Life Sciences, Technical University of Munich, 85354 Freising, Germany

Synucleinopathies are neurodegenerative diseases characterized by accumulation of misfolded α-synuclein (α-syn) inclusions in neuronal and/or glial cells. Despite differences in the underlying pathophysiology, synucleinopathies often are misdiagnosed, especially in early stages, due to the overlapping clinical symptoms [1].

Recently, our group has shown that α-syn measured in both neuronal extracellular vesicles (nEVs) and oligodendroglial EVs (oEVs) in the same samples, and in particular the oEV:nEV α-syn concentration ratio, yielded a discriminative model distinguishing multiple system atrophy (MSA) from healthy controls (HC) or Parkinson’s disease (PD) with high sensitivity and specificity. In contrast, the separation between PD and HC was moderate [2].

In this study, using remaining samples from the previous study, we evaluated whether adding nEV and oEV pS129-α-syn, total tau, tau phosphorylated at Thr 181 (pT181-tau), and/or serum neurofilament light chain (NfL) to the previously measured α-syn might improve the diagnostic power. Patient information is shown in Additional file 1: Tables S1–S3. Most results are presented as log-transformed values to facilitate normal distribution of the data. Non-transformed values are summarized in Additional file 1: Table S4.

The level of pS129-α-syn in normal brain is ~ 4% of total α-syn and may increase to ~ 90% in Lewy bodies [3]. pS129-α-syn is also enriched in glial-cytoplasmic inclusions though to a lesser degree [4], suggesting that its concentrations in CNS-originating EVs may help distinguish among the groups.

Measurement of pS129-α-syn in 32 HC, 46 PD, and 30 MSA samples showed that in many cases its concentration constituted a small fraction of total α-syn (Additional file 1: Fig. S1a). The nEV pS129-α-syn concentrations decreased in the order HC > PD > MSA, yet the differences were statistically insignificant (Fig. 1a). In contrast, the oEV concentrations of pS129-α-syn increased in the same order, HC < PD < MSA, and were significantly higher in both disease groups (Fig. 1a). Thus, pS129-α-syn concentration in nEVs was not affected by synucleinopathy, whereas in oEVs it was increased in MSA and in a subgroup of patients with PD compared to HCs.

**Fig. 1:**
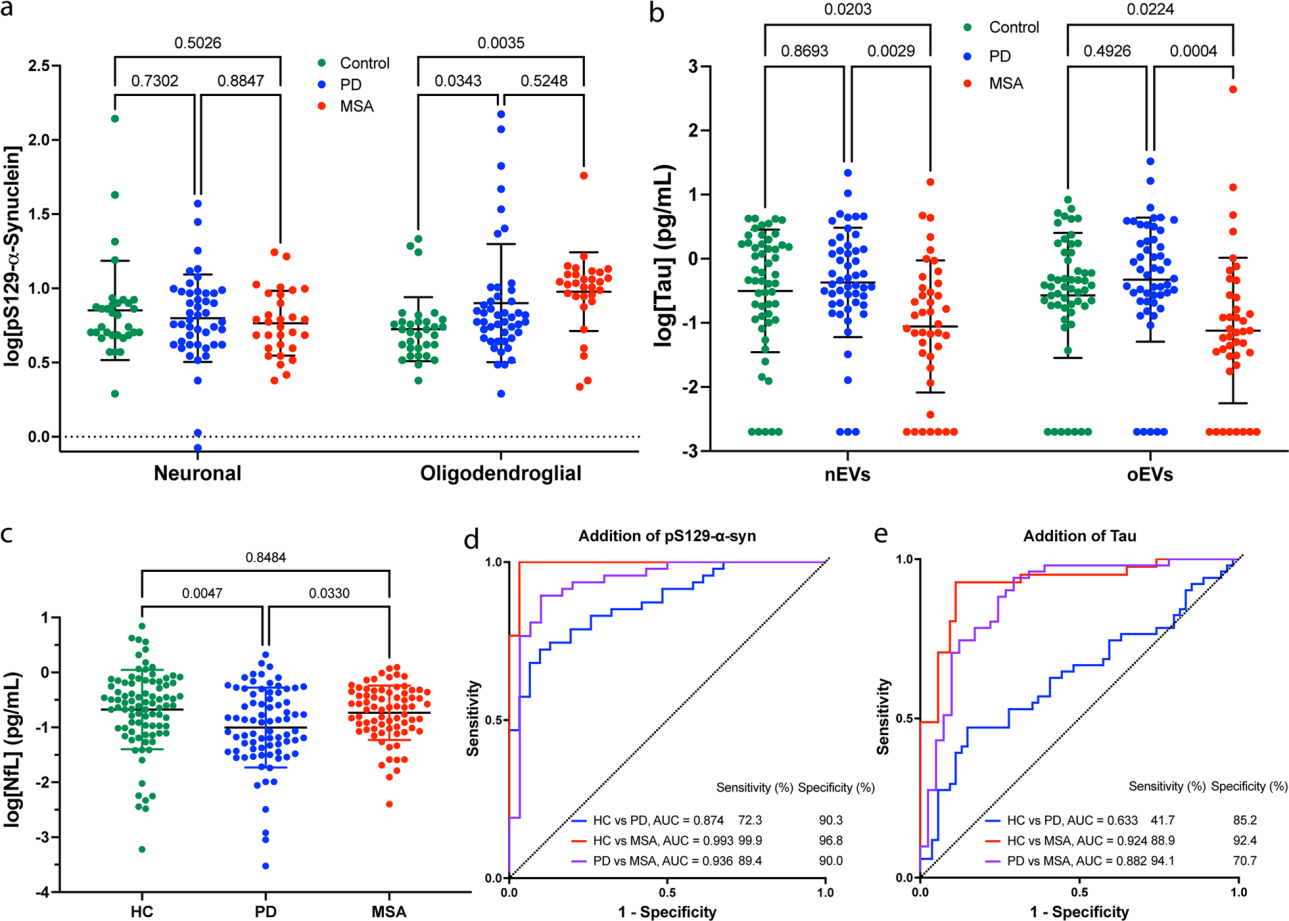
Biomarker analysis in HC, PD, and MSA. **a**, **b** pS129-α-Syn and Tau concentrations were measured using ECLIA, log-transformed, and analyzed by a mixed-effect analysis with post-hoc Tukey test. Data are represented as mean ± SD. Values below the lower limit of detection were imputed as the minimum value divided by 2. **c** NfL concentrations were measured using Simoa, log-transformed, and analyzed by a one-way ANOVA with post-hoc Tukey test. Data are represented as mean ± SD. **d**, **e** ROC analyses of biomarker concentrations using a multinomial logistic regression model with LASSO variable selection (Additional file 1: Fig. S3). The model included nEV α-syn concentration, oEV:nEV α-syn concentration ratio, total EV concentration in the sample and **d** oEV pS129-α-syn or **e** oEV tau concentration. The presented models do not include NfL

The oEV:nEV pS129-α-syn ratio increased similarly in the order HC < PD < MSA (Additional file 1: Fig. S1b). In most patients with MSA this ratio was > 1, as reported previously for total α-syn [2]. However, in the PD group roughly equal numbers of samples had a ratio < 1 or > 1. Consequently, unlike the oEV:nEV total α-syn ratio, the oEV:nEV pS129-α-syn ratio separated the groups only moderately.

Genome-wide association and other genetic studies have revealed a strong link of the MAPT gene to PD [5] and tau aggregates have been observed in ~ 50% of PD brains. In contrast, tau pathology is rare in MSA. Thr 181 is a common tau phosphorylation site in pathological conditions and CSF pT181-tau has been used as a biomarker for PD and atypical parkinsonian disorders [6].

Total tau was measured in 54 HC, 51 PD, and 41 MSA samples. The tau concentrations were approximately an order of magnitude lower than those of α-syn (Additional file 1: Table S4). A few samples yielded signal below the lower limit of detection, which was imputed as the minimum/2 value (Fig. 1b). Analysis without the imputed values did not change the significance or direction of the results. The average concentration in both nEVs and oEVs was significantly lower in the MSA group compared to HC and PD, in agreement with the scarce observation of tau pathology in MSA. The differences remained when the data were adjusted for age. The HC and PD groups had similar tau concentrations in both nEVs and oEVs. The differences between nEVs and oEVs were statistically insignificant in all three groups (Fig. 1b), suggesting that measurement of tau in one type of EV is sufficient.

In our first attempt to measure pT181-tau we isolated nEVs and oEVs from 9 HC, 17 PD, and 12 MSA samples. Unfortunately, pT181-tau was detectable only in 6 of the 76 EV lysates, suggesting that the pT181-tau levels were a small fraction of the total tau concentration and that at the present assay-sensitivity level this analyte is not useful for distinguishing among HC, PD, and MSA. Alternatively, the low levels may be due to sample age.

CSF NfL is a useful marker of neuroaxonal injury and neurodegeneration and plasma NfL concentrations have been shown to correlate well with CSF levels [7]. Significantly higher plasma NfL concentrations have been reported in MSA compared to HC and PD [8]. Therefore, we tested whether similar differences could be detected in our cohort, though we used serum rather than plasma. NfL concentrations in serum and plasma have been reported to correlate well in patients with multiple sclerosis [7] though we are not aware of a similar comparison in patients with synucleinopathies.

Here, as the volume needed for direct measurement of NfL (20 μl) is ~ 10% of the volume needed for EV isolation, we measured serum NfL in most of the samples—88 HC, 79 PD, and 74 MSA, using a Simoa assay to match most of the current literature. We found a significantly lower average NfL concentration in the PD group compared to HC and MSA (Fig. 1c), even after the data were adjusted for age. The lower concentration of NfL in PD compared to HC samples was in agreement with a recent paper by Chen et al. [9] who found that the difference was statistically significant only in females. Consistently, in our cohort, we found lower NfL concentrations in both PD and MSA samples from females but not from males (Additional file 1: Fig. S2).

Finally, we used the same statistical approach as in [2] to construct new models (Additional file 1: Fig. S3) including both the previously selected parameters (nEV α-syn concentration, oEV:nEV α-syn concentration ratio, and total EV concentration) and the new biomarker measurements, to evaluate whether pS129-α-syn or tau might improve the separation when added separately, as the number of samples in which both pS129-α-syn and tau were measured was too small in this study to allow meaningful evaluation of both together. NfL was measured in most of the samples and therefore we tested it in combination with pS129-α-syn or tau.

For the subset of samples with pS129-α-syn measurements, addition of oEV pS129-α-syn concentration to the previous parameters in the ROC analysis resulted in improved predictions (Akaike information criterion [AIC] = 136.1 vs 138.7; AIC change = − 2.6). The improved model separated between the PD and HC groups with AUC = 0.874 (95% CI 0.82–0.96), MSA and HC with AUC = 0.993 (95% CI 0.98–0.99), and PD and MSA with AUC = 0.936 (95% CI 0.88–0.99) (Fig. 1d, Additional file 1: Table S5). Addition of NfL did not improve the separation in this sample subset.

For the tau measurement subset, addition of oEV tau did not improve the model predictions significantly (Fig. 1e, Additional file 1: Table S5). Adding NfL to oEV tau did improve the model predictions (AIC change = − 2.2) and the AUC value for separation between HC and PD increased from 0.633 to 0.720 (*P* = 0.055). α-Syn concentration in both nEVs and oEVs in all the groups combined correlated with oEV pS129-α-syn (*r*_nEV_ = 0.38, *r*_oEV_ = 0.35; *P* < 0.0001) but not with nEV pS129-α-syn. Analysis in each group showed no significant correlations in the control group between nEV or oEV total α-syn and nEV or oEV pS129-α-syn, indicating that this group did not contribute to the observed correlations. In the PD group, both nEV and oEV α-syn correlated positively with oEV pS129-α-syn (*r*_nEV_ = 0.37, *P* = 0.010; *r*_oEV_ = 0.33, *P* = 0.025). Similarly, positive correlations were found for the MSA group (*r*_nEV_ = 0.38, *P* = 0.037; *r*_oEV_ = 0.38, *P* = 0.036). None of the biomarkers correlated with disease duration or disease progression as assessed by Unified Parkinson’s Disease Rating Scale-III, Unified Multiple System Atrophy Rating Scale, or Hoehn and Yahr scale.

Our study is the first to measure pS129-α-syn in nEVs and oEVs and assess tau concentrations in these types of EVs in patients with MSA. Though the results need validation in larger cohorts, the data support measurement of these and additional biomarkers in CNS-originating EVs toward constructing a panel comprising at a minimum nEV α-syn, oEV:nEV α-syn ratio, oEV pS129-α-syn, and total EV concentration for improved diagnosis of parkinsonian disorders.

## Supplementary Information

**Additional file 1:** **Materials and Methods**. **Figure S1.** Ratios between pS129-α-syn and total α-syn concentrations and between pS129-α-syn concentrations in oEVs and nEVs. **Figure S2**. NfL concentrations are lower in the disease groups in females but not males. **Figure S3**. Selection of LASSO Coefficients. **Table S1.** Demographic and clinical characteristics of the patients whose samples were used for pS129-α-syn measurements. **Table S2.** Demographic and clinical characteristic of the patients whose samples were used for total tau measurements. **Table S3.** Demographic and clinical characteristic of the patients whose samples were used for neurofilament light chain (NfL) measurements. **Table S4.** Serum/plasma nEV and oEV biomarker measurements for the HC, PD, and MSA groups. **Table S5.** Multinomial logistic regressions with LASSO variable selection for separation among the HC, PD, and MSA groups.

## Data Availability

All the data generated or analyzed during this study are included in the published article.

## Abbreviations

nEVs: Neuronal EVs
NfL: Neurofilament light chain
oEVs: Oligodendroglial EVs
