# Supplementary material for: Toward a biomarker panel measured in CNS-originating extracellular vesicles for improved differential diagnosis of Parkinson’s disease and multiple system atrophy
Source: Transl Neurodegener. 2023 Mar 20;12:14. doi: 10.1186/s40035-023-00346-0 (PMC10026428; doi:10.1186/s40035-023-00346-0)
Supplement: Supplementary file 1 — Additional file 1: Materials and Methods. Figure S1. Ratios between pS129-α-syn and total α-syn concentrations and between pS129-α-syn concentrations in oEVs and nEVs. Figure S2. NfL concentrations are lower in the disease groups in females but not males. Figure S3. Selection of LASSO Coefficients. Table S1. Demographic and clinical characteristics of the patients whose samples were used for pS129-α-syn measurements. Table S2. Demographic and clinical characteristic of the patients whose samples were used for total tau measurements. Table S3. Demographic and clinical characteristic of the patients whose samples were used for neurofilament light chain (NfL) measurements. Table S4. Serum/plasma nEV and oEV biomarker measurements for the HC, PD, and MSA groups. Table S5. Multinomial logistic regressions with LASSO variable selection for separation among the HC, PD, and MSA groups. [file 40035_2023_346_MOESM1_ESM.docx]

**Toward a Biomarker Panel measured in CNS-originating Extracellular Vesicles for Improved Differential Diagnosis of Parkinson’s Disease and Multiple System Atrophy**

**Supplementary Material**

*Hash Brown Taha^1,2^, Simon Hornung^1^*^†^*, Suman Dutta^1^, Leony Fenwick^1^, Otmane Lahgui^1^, Kathryn Howe^1^, Nour Elabed^1^, Irish Del Rosario^3^, Darice Y. Wong^1,10^, Aline Duarte Folle^3^, Daniela Markovic^4^, Jose-Alberto Palma^5^, Un Jung Kang^9^, Roy N. Alcalay^6,^* ^7^*, Miriam Sklerov^8^, Horacio Kaufmann^5^, Brent L. Fogel^1,10,11^, Jeff M. Bronstein^1,12^, Beate Ritz^3,12^ and Gal Bitan^1,12,13*^*

^1^Department of Neurology, David Geffen School of Medicine, University of California Los Angeles, Los Angeles, CA, 90095, USA

^2^Department of Integrative Biology & Physiology, University of California Los Angeles, Los Angeles, CA, 90095, USA

^3^Department of Epidemiology, University of California Los Angeles Fielding School of Public Health, Los Angeles, CA, 90095, USA

^4^Department of Medicine Statistics Core, Division of General Internal Medicine and Health Services Research, University of California Los Angeles, Los Angeles, CA, 90095, USA

^5^Department of Neurology, Dysautonomia Center, New York University School of Medicine, New York, NY, 10016, USA

^6^Department of Neurology, Taub Institute for Research on Alzheimer’s Disease and the Aging Brain, Columbia University, New York, NY, 10032, USA

^7^Department of Neurology, Tel Aviv Sourasky Medical Center, Tel Aviv, Israel

^8^Department of Neurology, University of North Carolina School of Medicine, Chapel Hill, NC, 27599, USA

^9^Department of Neurology, The Marlene and Paolo Fresco Institute for Parkinson’s and Movement Disorders, New York University School of Medicine, New York, NY, 10016, USA

^10^Department of Human Genetics, David Geffen School of Medicine, University of California Los Angeles, Los Angeles, CA, 90095, USA

^11^UCLA Clinical Neurogenomics Research Center, David Geffen School of Medicine, University of California, Los Angeles, CA, 90095, USA

^12^Brain Research Institute, University of California, Los Angeles, CA, 90095, USA

^13^Molecular Biology Institute, University of California Los Angeles, Los Angeles, CA, 90095, USA

^†^Present address: Division of Peptide Biochemistry, TUM School of Life Sciences, Technical University of Munich, 85354 Freising, Germany

*To whom correspondence should be addressed:

Gal Bitan

Department of Neurology, David Geffen School of Medicine at UCLA, 635 Charles E. Young Drive South/Gordon Neuroscience Research Building 451, Los Angeles, CA 90095

E-mail: gbitan@mednet.ucla.edu

Phone: 310-206-2082

**Materials and Methods**

*Patient samples*

All the samples used were subsets of those described previously ([1](#_ENREF_1)). The determination of diagnosis and methods of sample collection and preparation were described previously. As the number of leftover samples from the previous study was limited, we combined the discovery and validation cohorts described previously ([1](#_ENREF_1)). Due to the limited sample volumes available, in most samples we measured NfL and either pS129-α-syn or tau. Therefore pS129-α-syn or tau were added to the discriminative model separately but not together. As this was an exploratory study, the samples were randomized into the different measurement groups without attempting to control for differences in the UPDRS scores or other parameters. Demographic and clinical characteristics and the number of samples analyzed per group for each biomarker are summarized in Supplemental Tables 1–3. All the biomarker data have been normalized to the total protein concentration measured using a BCA assay.

*EV isolation and immunocapture*

To remove intact cells and cell-debris, samples were centrifuged at 4 °C for 10 min at 3,400 *g*. Further isolation of nEVs and oEVs was done essentially as described previously ([1](#_ENREF_1)) except that before EV isolation, plasma samples were treated with 2 μl of a thrombin solution (Fisher Scientific, catalog no. ICN15416301) for 5 min with gentle agitation at room temperature. Fibrin was precipitated by centrifugation at 9,400 *g* for 5 min at room temperature. This treatment essentially converts the plasma into serum allowing analyzing all the samples together. Experimenters were blinded to the diagnosis, demographic data, or any other identifying information.

## *CD81 measurement*

The exosome concentration was estimated using the ExoELISA Ultra CD81 assay (System Biosciences) following the manufacturer’s instructions as described previously ([1](#_ENREF_1)).

## *pS129-α-syn assay*

We used a new in-house-developed electrochemilumiscence ELISA (ECLIA) based on Meso Scale Discovery (MSD)’s platform. The assay has been described in detail in ([2](#_ENREF_2)).

*Total tau and pT181-tau measurement*

Total tau and pT181-tau were measured using S-PLEX Human Total Tau or pT181-tau kits, respectively (MSD). Briefly, 50 μL of biotinylated human tau or pT181-tau capture antibodies were added to single-small spot streptavidin-coated wells and incubated at room temperature (RT) with shaking for 1 h at 700 rpm. After washing the plate using 150 μL wash buffer (WB: 0.05% (v/v) Tween-20 in 1X PBS, pH 7.4) per well thrice, 25 μL of blocking buffer and either sample or calibrator were added to each well and allowed to incubate at RT for 1.5 h with shaking at 700 rpm. The wells again were washed thrice using the WB, and 50 μL TURBO-BOOST human tau detection antibody was added to each well and incubated for 1 h with shaking at 700 rpm. The plate was washed thrice using the WB and incubated with 50 μL enhancing solution at RT for 30 min with shaking at 700 rpm. Each well then was washed thrice with WB and 50 μL of TURBO-TAG enhancing solution was added to each well and incubated at 27 °C for 1 h with shaking at 700 rpm. Lastly, the plate was washed thrice using WB, 150 μL of MSD Gold Read Buffer B was added to each well, and the plate was read immediately using a QuickPlex SQ 120 instrument. The data were analyzed using Discovery Workbench 4.0 and quantified with reference to freshly prepared total tau or pT181-tau standard curves.

The reported lower limit of detection (LLoD) of the S-PLEX total tau kit is 0.012 pg/mL and that of the pT181-tau kit is 0.077 pg/mL. In our hands, the experimental LLoDs were 0.014 and 0.019 pg/mL, respectively, for these assays. The intra- and inter-assay coefficients of variation (CVs) for total tau measurements were 9.2% and 2.9%, respectively. We did not determine CVs for the pT181-tau kit. One total tau sample was excluded from the analysis of the oEV MSA group because it was 39 standard deviations above the mean.

## *NfL measurement by Single Molecule Array (Simoa)*

NfL was measured directly in the serum samples. Samples were thawed once for aliquoting before measurements, diluted 1:20 (20 μL) in PBS (380 μL) containing a protease and phosphatase inhibitor cocktail (Halt^TM^ Protease and Phosphatase Inhibitor Cocktail, ThermoFisher Scientific), and analyzed by UCLA’s Immunogenetics Center using Simoa NfL kits (Quanterix) on an HD-X Analyzer (Quanterix).

*Statistical analysis*

One-way comparisons for the biomarkers between the groups were conducted using the Kruskal-Wallis test followed by a post hoc Tukey test. Covariate analyses were conducted using ANCOVA. Sample concentrations that were below the LLoD of the assay were imputed as the minimum value divided by 2 ([3](#_ENREF_3)). Correlations across individual biomarkers or between biomarkers and clinical test scores were evaluated using Spearman’s method. A multinomial logistic regression model with Least Absolute Shrinkage and Selection Operator (LASSO) variable selection was used to select the best variables to include in logistic models followed by receiver operating characteristic (ROC) analysis (Figure S3). The 95% confidence intervals of areas under the curve (AUC) were computed using the bootstrap method (n = 500). Sensitivity and specificity were estimated at the best threshold, defined as the value of the linear predictor in the logistic model which maximized the unweighted sum of the sensitivity and specificity using ROC models for each pairwise combination of groups (HC vs. PD, HC vs. MSA and PD vs. MSA). Prediction error was compared using the Akaike Information Criterion (AIC). Analyses were performed using Prism 9.4 (GraphPad) or R version 4.0.2 (Copyright © 2020 The R Foundation for Statistical Computing). Most results are presented in the figures as log-transformed values to improve obtaining normal distributions and facilitate statistical analysis. Non-transformed values are summarized in Table S4.


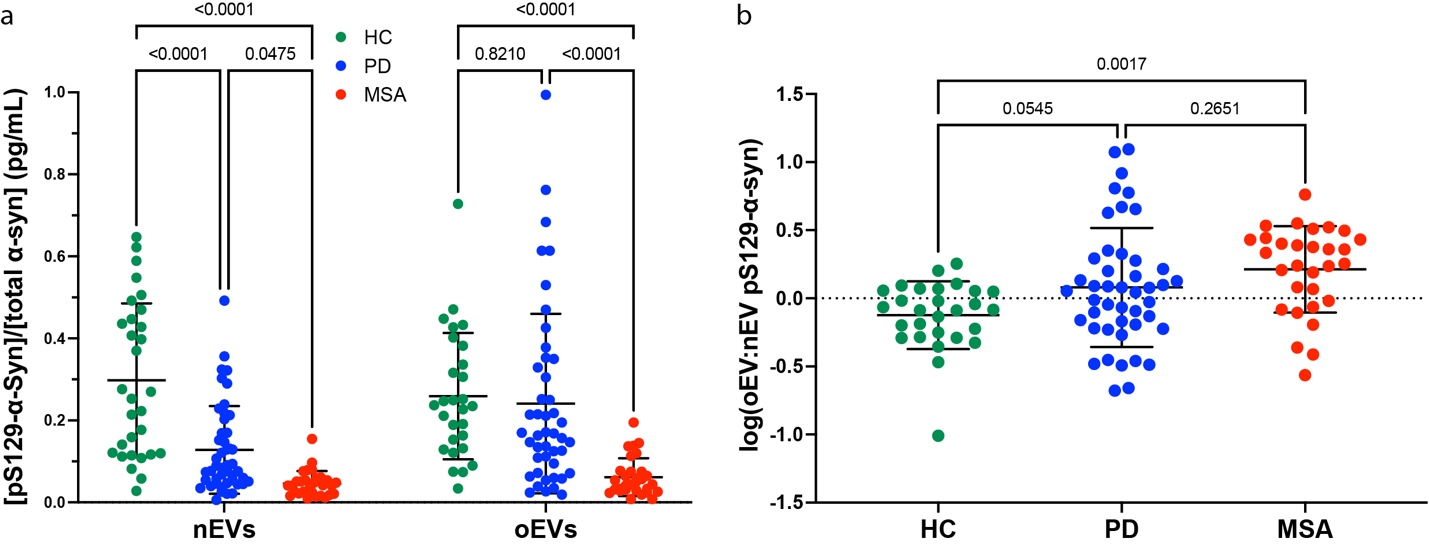


**Supplemental Figure S1. Ratios between pS129-α-syn and total α-syn concentrations and between pS129-α-syn concentrations in oEVs and nEVs.** a) Ratio between the concentrations of pS129-α-syn and total α-syn in each type of EV. P-values were calculated using a mixed model in Prism 9.4 (GraphPad). The apparent decrease in the order HC > PD > MSA reflects the fact that the differences in total α-syn concentration are larger than those in pS129-α-syn. b) Ratio between pS129-α-syn measured in oEVs and nEVs in each sample. P-values were calculated using a one-way ANOVA.

**Supplemental Figure S2. NfL concentrations are lower in the disease groups in females but not males.** NfL was measured directly in serum samples. P-values were calculated using a two-way ANOVA.


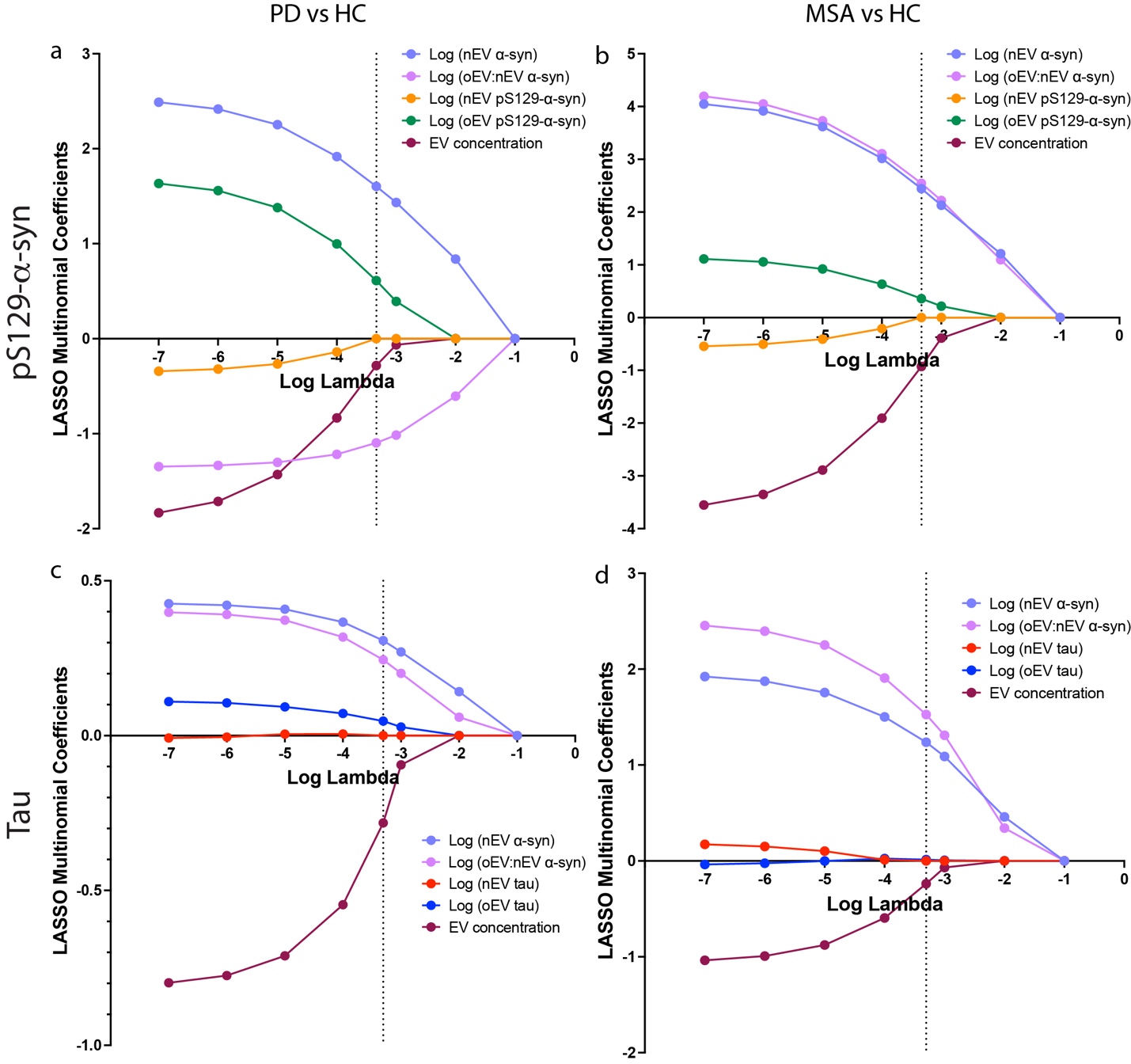


**Supplemental Figure S3. Selection of LASSO Coefficients.** The lambda parameter was chosen using 10-fold cross validation to minimize the prediction error (deviance statistic). The LASSO multinomial coefficients are shown as a function of lambda for pS129-α-syn in the comparison of PD vs HC (A) and MSA vs HC (B). The LASSO multinomial coefficients are shown as a function of lambda for tau in the comparison of PD vs HC (C) and MSA vs HC (D). A vertical line in each panel denotes the best lambda value. At the best value of lambda, four predictors remained in the model each case, whereas other coefficients dropped to zero.

**Supplemental Table 1.** Demographic and clinical characteristics of the patients whose samples were used for pS129-α-syn measurements.

| Variable | All Samples | Control | PD | MSA (C:P) |
| --- | --- | --- | --- | --- |
| Total number | 109 | 32 | 47 | 30 (26:4) |
| Age (range)^a^ | 66.8 ± 11.6 (35-90) | 60.2 ± 13.1(35-84) | 71.6 ± 9.8 (37-90) | 62.7 ± 8.2 (46-79) |
| Sex (male:female) | 58:51 | 17:15 | 28:19 | 13:17 |
| Disease Duration (range)^a^ |  |  | 8.1 ± 5.0  (0-19) | 4.4 ± 2.6 (0-10) |
| Race/ethncity^b^ | As^c^ - 8 | As – 1 | As – 0 | As – 7 |
|  | B - 2 | B - 0 | B - 1 | B - 1 |
|  | H - 1 | H - 0 | H - 0 | H - 1 |
|  | HN - 14 | HN - 6 | HN - 8 | HN - 0 |
|  | NA - 2 | NA - 1 | NA - 1 | NA - 0 |
|  | ND - 1 | ND - 0 | ND - 0 | ND - 1 |
|  | W - 81 | W - 24 | W - 37 | W - 20 |
| UPDRS^d^ (range) |  |  | 25.1 ± 15.6 (0-59) |  |
| H&Y^d^ (range) | 2.7 ± 1.1 (1-5) |  | 2.5 ± 1.0  (2-5) | 3.8 ± 1.0 (1-5) |
| MMSE^d^ (range) | 26.4 ± 6.9 (0-30) |  | 26.3 ± 6.4  (0-30)^e^ | 26.5 ± 9.3 (0-30) |

^a^Mean ± SD. ^b^As — Asian; B — Black; H — Hispanic; HN — Hispanic non-White; NA — Native American; ND — non-disclosed; W — White. ^c^Korea — 2; Philippines — 1; Taiwan — 1; Vietnam —1; Undefined Asian — 3. ^d^UPDRS — Unified Parkinson’s disease rating scale; H&Y —Höhn and Yahr rating scale; MMSE —Mini-Mental State Examination. ^e^Converted from Montreal Cognitive Assessment (MoCA) according to Lawton et al. ([4](#_ENREF_4)).

**Supplemental Table 2.** Demographic and clinical characteristic of the patients whose samples were used for total tau measurements.

| Variable | All Samples | Control | PD | MSA (C:P) |
| --- | --- | --- | --- | --- |
| Total number | 153 | 54 | 51 | 48 (21:3) |
| Age (range)^a^ | 67.1 ± 10.6  (35-90) | 64.8 ± 11.0  (35-88) | 71.1 ± 10.0  (40-90) | 63.7 ± 8.6  (47-79) |
| Sex (male:female) | 58:51 | 35:16 | 35:16 | 31:17 |
| Disease Duration (range)^a^ |  |  | 8.7 ± 4.6 (1-20) | 5.7 ± 5.2 (1-26) |
| Race/ethncity^b^ | As^c^ - 4 | As – 1 | As – 1 | As – 2 |
|  | B - 3 | B - 0 | B - 1 | B - 2 |
|  | H - 15 | H - 10 | H - 5 | H - 0 |
|  | HN - 8 | HN - 4 | HN - 4 | HN - 0 |
|  | NA - 3 | NA - 0 | NA - 3 | NA - 0 |
|  | ND - 4 | ND - 4 | ND - 0 | ND - 0 |
|  | W - 92 | W - 35 | W - 37 | W - 20 |
| UPDRS^d^ (range) |  |  | 12.6 ± 1.0 (0-5) |  |
| H&Y^d^ (range) | 2.78 ± 1.1 (0-5) |  | 2.5 ± 1.0 (2-5) | 4.5 ± 0.6 (4-5) |
| MMSE^d^ (range) | 26.1 ± 5.7 (0-30) |  | 26.3 ± 5.0  (1-30)^e^ | 24.9 ± 8.4  (0-30) |

^a^Mean ± SD. ^b^As — Asian; B — Black; H — Hispanic; HN — Hispanic non-White; NA — Native American; ND — non-disclosed; W — White. ^c^Korea — 0; Philippines — 0; Taiwan — 0; Vietnam —0; Undefined Asian — 4. ^d^UPDRS — Unified Parkinson’s disease rating scale; H&Y —Höhn and Yahr rating scale; MMSE —Mini-Mental State Examination. ^e^Converted from Montreal Cognitive Assessment (MoCA) according to Lawton et al. ([4](#_ENREF_4)).

**Supplemental Table 3.** Demographic and clinical characteristic of the patients whose samples were used for neurofilament light chain (NfL) measurements.

| Variable | All Samples | Control | PD | MSA (C:P) |
| --- | --- | --- | --- | --- |
| Total number | 241 | 88 | 79 | 74 (56:18) |
| Age (range)^a^ | 67.0 ± 10.2 (35-90) | 66.4 ± 10.0 (35-88) | 72.0 ± 10.3  (37-90) | 62.5 ± 7.7 (46-79) |
| Sex (male:female) | 125:116 | 40:48 | 51:27 | 34:40 |
| Disease Duration (range)^a^ | 6.9 ± 4.4 (0-26) |  | 8.6 ± 4.4 (0-20) | 5.1 ± 3.7 (0-26) |
| Race/ethncity^b^ | As^c^ - 16 | As – 2 | As – 1 | As –13 |
|  | B - 5 | B - 0 | B - 0 | B - 5 |
|  | H - 24 | H - 13 | H - 9 | H - 2 |
|  | HN - 10 | HN - 4 | HN - 6 | HN - 0 |
|  | NA - 8 | NA - 3 | NA - 5 | NA - 0 |
|  | ND - 7 | ND - 5 | ND - 0 | ND - 2 |
|  | W - 171 | W - 61 | W – 58 | W - 52 |
| UPDRS^d^ (range) |  |  | 24.4 ± 13.6 (0-50) |  |
| H&Y^d^ (range) | 2.9 ± 0.9 (0-5) |  | 2.4 ± 0.8 (0-5) | 3.6 ± 1.3 (1-5) |
| MMSE^d^ (range) | 28.0 ± 3.4 (0-30) |  | 27.0 ± 4.3  (0-30)^e^ | 28.3 ± 1.6 (24-30) |

^a^Mean ± SD. ^b^As — Asian; B — Black; H — Hispanic; HN — Hispanic non-White; NA — Native American; ND — non-disclosed; W — White. ^c^Korea — 2; Philippines — 1; Taiwan — 1; Vietnam — 1; Undefined Asian — 11. ^d^UPDRS — Unified Parkinson’s disease rating scale; H&Y —Höhn and Yahr rating scale; MMSE —Mini-Mental State Examination. ^e^Converted from Montreal Cognitive Assessment (MoCA) according to Lawton et al. ([4](#_ENREF_4)).

**Supplemental Table 4.** Serum/plasma nEV and oEV biomarker measurements for the HC, PD, and MSA groups. All values are reported as median (CI).

| **Serum/plasma** | | | |
| --- | --- | --- | --- |
| Group | HC | PD | MSA |
| CD81 (particles/mL) | 6.19 × 10^10^ (96.5%) | 5.00 × 10^10^ (95.6%) | 4.10 × 10^10^ (95.8%) |
| NfL (pg/mL) | 262 (95.8%) | 85.7 (95.8%) | 232 (96.6%) |
| **Neuronal EVs** | | | |
| pS129-α-Synuclein (pg/mL) | 6.06 (98.0%) | 5.95 (96.0%) | 5.72 (95.7%) |
| Total tau (pg/mL) | 0.46 (96.0%) | 0.40 (97.1%) | 0.10 (96.5%) |
| **Oligodendroglial EVs** | | | |
| pS129-α-Synuclein (pg/mL) | 5.17 (98.0%) | 6.39 (96.0%) | 10.7 (95.7%) |
| Total tau (pg/mL) | 0.38 (96.0%) | 0.56 (97.1%) | 0.07 (98.3%) |

**Supplemental Table 5.** Multinomial logistic regressions with LASSO variable selection for separation among the HC, PD, and MSA groups. AUC — area under the curve

|  | Control vs. PD | | | Control vs. MSA | | | PD vs. MSA | | |
| --- | --- | --- | --- | --- | --- | --- | --- | --- | --- |
|  | AUC | Sensitivity | Specificity | AUC | Sensitivity | Specificity | AUC | Sensitivity | Specificity |
| Dutta et al. Acta Neuropathol. 2021 – Discovery Cohort^1^ | 0.762 | 60.8% | 85.7% | 0.961 | 96.7% | 89.8% | 0.928 | 82.4% | 93.3% |
| Dutta et al. Acta Neuropathol. 2021 – Validation Cohort | 0.610 | 71.4% | 62.7% | 0.962 | 96.0% | 84.3% | 0.902 | 89.8% | 86.0% |
| Addition of oEV pS129-α-syn^2^ | 0.874 | 72.3% | 90.3% | 0.993 | 99.9% | 96.8% | 0.936 | 89.4% | 90.0% |
| Addition of oEV tau^2^ | 0.633 | 47.1% | 85.2% | 0.924 | 88.9% | 92.4% | 0.882 | 94.1% | 70.7% |
| ^1^The model was created based on the data in this cohort. New models were created using the previous total α-syn and total EV concentration results together with either oEV pS129-α-syn or oEV tau data. | | | | | | | | | |

**References**

1. Dutta S, Hornung S, Kruayatidee A, Maina KN, Del Rosario I, Paul KC, et al. α-Synuclein in blood exosomes immunoprecipitated using neuronal and oligodendroglial markers distinguishes Parkinson's disease from multiple system atrophy. Acta Neuropathol. 2021;142(3):495-511.

2. Dutta S, Hornung S, Taha HB, Biggs K, Siddique I, Chamoun LM, et al. Development of a novel electrochemiluminescence ELISA for quantification of α- synuclein phosphorylated at Ser129 in biological samples. ACS Chem Neurosci. 2023:In press.

3. Succop PA, Clark S, Chen M, Galke W. Imputation of data values that are less than a detection limit. J Occup Environ Hyg. 2004;1(7):436-41.

4. Lawton M, Kasten M, May MT, Mollenhauer B, Schaumburg M, Liepelt-Scarfone I, et al. Validation of conversion between mini-mental state examination and montreal cognitive assessment. Mov Disord. 2016;31(4):593-6.
